# Longitudinal study of rural health workforce in five counties in China: research design and baseline description

**DOI:** 10.1186/1478-4491-11-17

**Published:** 2013-05-04

**Authors:** Huiwen Xu, Weijun Zhang, Xiulan Zhang, Zhiyong Qu, Xiaohua Wang, Zhihong Sa, Yafang Li, Shuliang Zhao, Xuan Qi, Donghua Tian

**Affiliations:** 1 China Institute of Health, School of Social Development and Public Policy, Beijing Normal University, 19 Xinjiekou Wai Street, Haidian District, Beijing, 100875, China

**Keywords:** Rural health workforce, Human resources for health, Village doctors, Barefoot doctors, China

## Abstract

**Background:**

The village doctors have served rural residents for many decades in China, and their role in rural health system has been highly praised in the world; unfortunately, less attention has been paid to the health workforce during the ambitious healthcare reform in recent years. Therefore, we conducted a longitudinal study to explore the current situation and track the future evolution of the rural healthcare workforce.

**Methods:**

The self-administered structured Village Clinic Questionnaire and Village Doctor Questionnaire, which were modified from the official questionnaires of the Ministry of Health, were constructed after three focus groups, in-depth interviews in Hebei Province, and a pilot survey in Sichuan Province. Using a stratified multistage cluster sampling process, we gathered baseline data for a longitudinal survey of village doctors, village clinics from Changshu County, Liyang County, Yongchuan District, Mianzhu County, and Jingning County in China in 2011. Well-trained interviewers and strict procedures were employed to ensure the quality of this survey. Descriptive and correlation analyses were performed with Stata 12.0.

**Results:**

After four months of surveying, 1,982 Village Doctor Questionnaires were collected, and the response rate was 88.1%. There were 1,507 (76.0%) male and 475 (24.0%) female doctors, with an average age of 51.3 years. The majority of village doctors (58.5%) practiced both western medicine and Traditional Chinese Medicine, and 91.2% of the doctors received their education below college level. Their practice methods were not correlated with education level (*P* = 0.43), but closely related to the way they obtained their highest degree (that is, prior to starting work or as on-the-job training) (*P* < 0.01). The mean income of the village doctors was 1,817 (95% CI 1,733 to 1,900) RMB per month in 2011; only 757 (41.3%) doctors had pensions, and the self-reported expected pension was 1,965 RMB per month.

**Conclusions:**

Village doctors in rural China are facing critical challenges, including aging, gender imbalance, low education, and a lack of social protection. This study may be beneficial for making better policies for the development of the health workforce and China’s healthcare reform.

## Background

China has undergone ambitious and systematic healthcare reform since the central government established a series of healthcare policies in 2009 [1] to meet the demands of its 1.3 billion citizens for an equitable, affordable, and efficient healthcare system. The reform efforts over the previous 3 years have put priorities on the financing of public health and primary health care, the subsidization of universal health insurance coverage, the establishment of National Essential Drugs List, and public hospital reform [2]. These issues are closely related to the human resources for health, and the new policies affect medical service practice, productivity, and time allocation for public health and clinical service. For example, public health programs have been launched, and the numbers of patients seeking medical service have greatly increased due to the ongoing universal health coverage. On the other hand, the healthcare workers will also influence the progress and implementation of the reforms [3], and the shortage and misdistribution of healthcare workforces are major obstacles to strengthening the public healthcare and the primary health care system and to achieving the policy goals of health care reform [2]. Therefore, more attention should be paid to the development of the healthcare workforce [4], especially in rural areas, where the human resources are typically under-provided in both quantity and quality because of low salary, uncomfortable work environment, and poor education for young children [5-7].

In regard to the healthcare workforce in China, the most well-known and widely discussed issue might be the barefoot doctors in rural China [8-13], which has served as an example of solving shortages of medical services in rural areas [14]. In 1968, the barefoot doctors program became a national policy and obtained high political priority in order to solve the substantial shortage of healthcare workers in rural China [15]. At the same time, the quantity of barefoot doctors increased dramatically as they were allowed to provide medical services after a short period of training at a county or community hospital [14]. By the 1970s, there were approximately 5 million rural healthcare workers, including 1.8 million barefoot doctors, 3.5 million hygienists, and 0.7 million midwives [16]. During that time, immunization, maternal health care, and other public health services, together with medical services were delivered by barefoot doctors [14]. Most barefoot doctors practiced a mixture of basic western medicine and Traditional Chinese Medicine (TCM). Barefoot doctors, together with the Cooperative Medical Scheme, which covered the cost of medical services, ensured the success in the prevention of communicable diseases, reduced the infant mortality rate, and lengthened life expectancy in rural areas of China [17].

Since the 1980s, China has undergone a large and complex economic and social transition [18-22], and the collective economy and Cooperative Medical Scheme collapsed in rural China, which resulted in a decrease in numbers of barefoot doctors [14]. Concurrently, the Ministry of Health (MOH) started to streamline the numbers of barefoot doctors and to improve their quality [16], and in 1985 the title ‘barefoot doctor’ was abandoned; about 0.64 million doctors who passed the qualifying examination obtained a certificate as a village doctor [14,23].

To further improve the quality of village doctors, the central government published a series of policies beginning in the 1990s. In 1991, the MOH released the ‘1991-2000 National Education Plan of Village Doctors,’ which required 80% of village doctors to reach the requirements of Systematization and Normalization. In 2002, the ‘2001-2015 Health Workforce Development Outlines’ were published with specific goals: village doctors should obtain an education degree of secondary school or above, and 85% of village doctors should pass the exam and get the assistant doctor license by the end of 2015. The ‘Village Doctors Practitioners Regulation’ was released by the State Council in 2003, which particularly regulated the certification, practice, training and legal obligations of village doctors, establishing the first set of national regulations for village doctors. After the regulations were published, the number of village doctors quickly decreased to 0.8 million in 2003 [24]. As a result of the New Cooperative Medical Scheme, which was introduced in 2003 and continued the economic growth that began in the 1980s, the healthcare demands of rural residents dramatically increased. The number of healthcare workers in rural areas was subsequently increased, including 1 million village doctors and 65,895 hygienists, and there were about 1.3 rural healthcare workers (both village doctors and hygienists) per 1000 rural residents at the end of the year 2011 [24].

Although several national policies were established for village doctors, there were no special guidelines regarding their income or pension. In fact, since 1985, the village doctors have become private, for-profit practitioners [14], and most of their income was derived from their medical practice and nothing was derived from public health service. However, such situations have been changed since the healthcare reform began. In the reformed health system, village doctors have been required to deliver both public health and primary medical services to rural residents, and the Essential Drugs List was conducted in the village clinics, which required the village doctors to provide drugs with zero-profit [2]. To compensate the potential income loss of village doctors, the central government established three refund channels: the public health subsidies, the Essential Drugs List allowance, and a general outpatient fee. Hence, the new policy inevitably led to medical service behavior changes of village doctors. However, the outcome of the new policy is still unclear.

The studies on barefoot doctors began showing up in 1972 when there were several articles that introduced the development, training, payment, and job satisfaction of barefoot doctors [9,11-13,25]. Several other studies discussed the potential problems of barefoot doctors including decentralized training, low quality, job dissatisfaction [26], number, age, workload and income of village doctors [27,28], and even suggested the possibility of exporting the concept of barefoot doctors to other countries [29]. After the collapse of the collective economy and following the economic reform, the transition and training mechanisms of village doctors were studied [21,30,31], and the recent studies investigated the evolution [14,16] and the prescribing behavior of village doctors [32].

However, there are problems among these studies. Most of the studies were conducted decades ago, and as a result of the substantial social and economical changes in recent years, these studies do not describe the current status of village doctors; nor do most of these studies contain detailed data describing the characteristics of village doctors. Furthermore, the annual organization-based administrative survey conducted by the MOH only covers health institutions at the township level and above, and lack comprehensive information about village doctors [23,33].

Because the village doctors act as gatekeepers for the rural residents [24], they are likely to have a profound impact on China’s healthcare system and on the health status of rural residents. Consequently, we conducted a longitudinal study of rural health workforce project in China to identify the basic demographic characteristics (for example, age, sex, and education) and current situations (for example, income and training) of these healthcare workers and to track their future trends, particularly the impacts of healthcare reform on the village doctors. For further understanding of the healthcare workforce in rural areas, healthcare workers in township health centers (THCs) were also investigated. This study primarily focuses on the research design, including questionnaire construction, sampling procedure, data collection and statistical analysis; and the baseline characteristics of village doctors.

## Methods

The Rural Health Workforce Project in China is a longitudinal nationwide study conducted by the China Institute of Health projected from year 2011 to 2020, and three waves of data are expected to be collected in 2011, 2015, and 2020. The baseline data (first wave) were gathered from July to November in 2011.

### Questionnaires

China has its own organization-based annual survey on healthcare institutions, but this survey primarily focuses on townships and above, and does not cover the individual data regarding rural healthcare workers [24,33]. Based on the official questionnaires from the MOH, and after adding detailed individual information, personal history and attitude changes after the healthcare reform to the questionnaires, we constructed a self-administered, structured Village Clinic Questionnaire and a Village Doctor Questionnaire.

After the initial version of the questionnaires were determined, we performed in-depth interviews with the local health administrators, directors and healthcare workers of the THCs and the village doctors in Xiong County, Hebei Province, in February 2011. We held three focus groups that included sociologists, physicians, statisticians, economists, health policy researchers, and administrators from the MOH to discuss the structure and contents of the questionnaires, and we undertook a major revision in May 2011 after convening the focus groups. In July 2011, a pilot survey was conducted in Mianzhu County, Sichuan Province, and 40 village doctors, 10 doctors from 3 THCs were interviewed with the questionnaires by the interviewers from the China Institute of Health. We then modified some questions that did not meet our needs, and developed the final version of the questionnaires.

The Village Doctor Questionnaire was divided into five parts: the basic information (age, gender, year started working as a village doctor, education, the manner of obtaining their highest degree of certification, and whether or not the doctor served as a director of a village clinic); primary healthcare services, public health services, charges of per visit (including common acute diseases and chronic diseases); the knowledge of hypertension and diabetes; the treatment of village doctors (for example, basic medical income, change of income after health reform, pension); and the training (including time, place, institute, contents and self-assessment of required training). The options provided for basic information were consistent with the official statistics to ensure a comparison between the data from the China Health Statistics Yearbook and other official statistics data.

### Sampling

A multistage cluster sampling method was used in this study. At the first stage , we selected Jiangsu Province (southeast), Sichuan Province (southwest), Chongqing Municipality (middle-west), and Gansu Province (northwest) from the 34 provinces or municipalities of China; then we chose a county or district from each province or municipality: Changshu County from Jiangsu; Mianzhu County from Sichuan; Yongchuan District from Chongqing and Jingning County from Gansu. To seek a balance of geographical and economic distribution between eastern China and western China, Liyang County from Jiangsu Province was added as the sample county. As a result, we selected five counties in China, whose basic characteristics in 2010, with comparisons to the national standard, are shown in Table 1. Changshu and Lilyang were in the developed eastern China; Mianzhu and Yongchuan were in the developing middle-east China, with a moderate level of economic development; and Jinning, from underdeveloped western China, was one of the Nationally Designated Poor Counties. Although considerations of the geographic distribution and socioeconomic status were taken in the sampling processes, we should acknowledge that the initial two steps of sampling were not random, and our choice of those counties was mainly based on the former collaboration between local health bureau and China Institute of Health.

**Table 1:** The basic characteristics of the sampling counties

**County**	**GDP per capita (RMB)**	**Financial expenditure (100 million RMB)**	**Population (10 thousand)**	**Rural residents (%)**	**Hospital beds (per 1000 capita)**	**Doctors (per 1000 capita)**
Changshu^a^[34]	96,518.0	95.4	106.7	20.2	4.6	2.3
Liyang^a^[34]	56,784.0	33.5	78.2	19.8	2.9	1.6
Mianzhu [35]	24,533.0	5.9	51.3	74.3	4.9	1.4
Yongchuan [36]	8,191.0	6.0	36.6	43.1	11.9	3.9
Jingning [37]	3,711.0	1.2	46.5	90.4	3.0	1.2
Mean	37,947.4	28.4	63.9	41.7	4.8	2.0
China[38]	29,992.0	-	133,972.0	50.3	3.6	1.8

^a^Because of the similarities between Changshu and Liyang, the two counties are combined in this study. GDP, gross domestic product.

The target population covered all village clinics and village doctors from the sample counties, while doctors who were not at practice were excluded. We obtained a list of all village clinics and village doctors in each county. As shown in Table 2, there were 1,271 village clinics (403 from Changshu and Liyang, 198 from Mianzhu, 270 from Yongchuan, and 400 from Jingning) and 2,250 village doctors (780 from Changshu and Liyang, 400 from Mianzhu, 700 from Yongchuan, and 370 from Jingning) listed in the table. All village doctors were asked to complete the Village Doctor Questionnaire, and village doctors who were the directors of village clinics were also asked to complete the Village Clinic Questionnaire.

**Table 2:** The quantities and response rates of village clinics and village doctors

**County**	**Village clinics**			**Village doctors**		
	**N**	**n**	**%**	**N**	**n**	**%**
Changshu and Liyang	403	303	75.2	780	659	84.5
Mianzhu	198	190	96.0	400	368	92.0
Yongchuan	270	253	93.7	700	604	86.3
Jingning	400	367	91.8	370	351	94.9
Total	1,271	1,113	87.6	2250	1,982	88.1

### Data collection

We sent ten interviewers to each county, including five professional investigators from the China Institute of Health and five employees from local health bureaus who were trained by experienced researchers for one week. The guidelines for investigators were created to illustrate the research objectives, explain each question, and standardize the interview procedure.

An informed consent form was given to each participant before the interview. If the respondents agreed to participate in the survey, then our interviewers gave a detailed explanation for the questions, supervised the entire survey process, and provided further introductions to interviewees when necessary. Interviewees who could not finish the questionnaire independently received help from project members. After the questionnaires were completed, the survey inspector examined the collected questionnaires; if the answers were incomplete or missed, a follow-up telephone interview was conducted. Overall, 78 questionnaires were redone by telephone interview.

### Data analysis

Data entry was accomplished by Double Entry and Validation through EpiData 3.1 (The EpiData Association, Odense M, Denmark). Data analyses regarding the age, gender, education, practice methods, income, and anticipated pensions of village doctors were primarily descriptive, and correlation analyses were conducted to evaluate the relationship between education and practice methods. Statistical analyses were performed using Stata 12.0 (StataCorp LP, College Station, Texas, USA).

## Results

The numbers and the response rates of village clinics and village doctors are presented in Table 2. Overall, 1,113 village clinics and 1,982 village doctors completed the questionnaires. The response rates were 87.6% and 88.1%, respectively.

### The gender and age distributions of the village doctors

The gender distributions and mean age of the village doctors in the surveyed counties are shown in Table 3. Of the 1,982 village doctors, 1,507 (76.0%) were male, and 475 (24.0%) were female. The gender distributions were different in different counties: 231 (35.1%) females in Changshu and Liyang, 109 (29.6%) in Mianzhu, 113 (18.7%) in Yongchuan and 22 (6.3%) in Jingning. Similarly, the male-to-female ratio was 1.9 in Changshu and Liyang, 2.4 in Mianzhu, 4.4 in Yongchuan, and 15.0 in Jingning, which showed the highest ratio.

**Table 3:** The gender and age distributions of village doctors

**County**	**Male (%)**	**Female (%)**	**Male/Female**	**Mean age**
Changshu and Liyang	428(64.9)	231(35.1)	1.9	56.4
Mianzhu	259(70.4)	109(29.6)	2.4	52.5
Yongchuan	491(81.3)	113(18.7)	4.4	48.3
Jingning	329(93.7)	22(6.3)	15.0	45.7
Total	1,507(76.0)	475(24.0)	3.2	51.3

The average age of all the interviewed village doctors was 51.3 years: 53.4 years in Changshu and Liyang, 52.5 years in Mianzhu, 48.3 years in Yongchuan, and 45.7 years in Jingning. It was found that age difference between Changshu and Liyang and Jingning was almost 10 years, indicating that the village doctors were much older in the relatively wealthier counties.

### Medical service practiced by village doctors

In contrast to western countries, TCM has a long history in China and still plays an important role in the healthcare system. Hence, we asked the question: ‘What kind of medicine do you practice: western medicine, TCM, or a mixture of western medicine and TCM?’ to investigate the type of medicine practiced by the village doctors. Of the 1,982 respondents, 1,979 village doctors answered the question. As shown in Table 4, 761 (38.5%) chose western medicine, only 60 (3.0%) chose TCM, and 1,158 (58.5%) chose mixed methods. In Changshu and Liyang, 44.6% of village doctors practiced western medicine. The ratios of western medicine doctors in Mianzhu and Yongchuan were similar (41.0% versus 41.8%). However, only 65 (18.5%) village doctors in Jingning reported practicing western medicine.

**Table 4:** Practicing methods of village doctors

**County**	**Western medicine (%)**	**Traditional Chinese medicine (%)**	**Mixed methods (%)**	**Total (%)**
Changshu and Liyang	294(44.6)	4(0.6)	361(54.8)	659(100)
Mianzhu	151(41.0)	8(2.2)	209(56.8)	368(100)
Yongchuan	251(41.8)	34(5.7)	316(52.6)	601(100)
Jingning	65(18.5)	14(4.0)	272(77.5)	351(100)
Total	761(38.5)	60(3.0)	1,158(58.5)	1,979^a^(100)

^a^Three data points are missing.

### Village doctor education levels

We also asked two questions to investigate the education levels of the village doctors: ‘What is your highest education level: junior high school or less, high school, secondary school, junior college, and college or above?’ and ‘In what manner did you obtain your highest education level: prior to employment or as on-the-job training?’. Overall, the highest education level obtained by most village doctors (91.2%) was secondary school (3 years’ medical training after graduating from junior high school) or less. Only 5 of 1,982 village doctors obtained a college degree or higher. The education level in Jingning was slightly higher than the other counties, and the education levels in Changshu and Liyang were the lowest.

As shown in Table 5, 1,057 (57.1%) village doctors reported that their highest education degrees were obtained after beginning working as a village doctor, while 793 (42.9%) village doctors reported the opposite. This finding implied that more than half of the village doctors obtained their highest degree through on-the-job training.

**Table 5:** The education level of village doctors

**County**	**Junior high school or less (%)**	**High school (%)**	**Secondary school (%)**	**Junior college (%)**	**College or above (%)**	**Total (%)**
Changshu and Liyang	188(28.5)	98(14.9)	339(51.4)	32(4.9)	2(0.3)	659(100)
Mianzhu	97(26.2)	38(10.3)	194(52.4)	40(10.8)	1(0.3)	370(100)
Yongchuan	121(20.1)	34(5.7)	394(65.5)	52(8.6)	1(0.2)	602(100)
Jingning	35(10.0)	13(3.7)	257(73.2)	45(12.8)	1(0.3)	351(100)
Total	441(22.3)	183(9.2)	1,184(59.7)	459(8.5)	5(0.3)	1,982(100)

Table 6 and Table 7 show the results of the correlation analyses of the relationship between the education and the practice method. The highest education degree was not correlated with the practice method (χ^2^ = 8.03, df = 8, *P* = 0.43). However, the way of obtaining the highest degree of education was closely related to the practice method (χ^2^ = 34.14, df = 2, *P* < 0.01). Compared with village doctors who obtained their highest education degree by on-the-job training, those obtaining their highest degree before working as a doctor had a greater probability of practicing western medicine (46.3% versus 33.0%), a lower probability of practicing mixed methods (64.0% versus 51.0%), and a similar probability of practicing TCM (3.0% versus 2.7%).

**Table 6:** The relationship between education and practicing methods

**Practice methods**	**Highest education degree**					
	**Junior high school or less (%)**	**High school (%)**	**Secondary school (%)**	**Junior college (%)**	**College or above (%)**	**Total (%)**
Western medicine	171(38.9)	80(44.2)	453(38.4)	54(32.1)	2(40.0)	760(38.5)
Traditional Chinese medicine	9(2.1)	5(2.8)	40(3.4)	4(2.4)	0(0.0)	58(2.9)
Mixed methods	260(59.1)	96(53.0)	687(58.2)	110(65.5)	3(60.0)	1,156(58.6)
Total	440(100)	181(100)	1,180(100)	168(100)	5(100)	1,974^a^(100)

^a^Eight data points are missing.

**Table 7:** The relationship between education and practicing methods of village doctors

**Practice methods**	**When highest education degree is earned**		
	**On-the-job (%)**	**Prior to employment (%)**	**Total (%)**
Western medicine	347(33.0)	366(46.3)	713(38.7)
Traditional Chinese medicine	32(3.0)	21(2.7)	53(2.9)
Mixed methods	674(64.0)	403(51.0)	1,077(58.4)
Total	1,053(100)	790(100)	1,843^a^(100)

^a^139 data points are missing.

### Village doctors’ pensions and income

Table 8 shows the basic income and pensions of the village doctors. The basic income was mainly the profits from medical services and did not include the public healthcare subsidy from the government or incomes from the agricultural activities of those village doctors who also had farmlands. The mean basic income was 1,817 RMB per month: 2,333 RMB in Changshu and Liyang, 1,958 RMB in Yongchuan, 1,019 RMB in Mianzhu, and 988 RMB in Jingning.

**Table 8:** The income and pension of village doctors as RMB per month and percent

**County**	**Basic income (95% CI)**	**Doctors with pensions (%)**	**Expected pensions (95% CI)**
Changshu and Liyang	2,333(2,233 to 2,432)	359(56.8)	2,051(1,972 to 2,131)
Mianzhu	1,019(900 to 1,139)	8(2.3)	1,591(1,509 to 1,672)
Yongchuan	1,958(1,763 to 2,152)	381(66.8)	2,420( 2,323 to 2,516)
Jingning	988 (808 to 1,168)	9(3.2)	1,177(1,081 to 1,272)
Total	1,817 (1,733 to 1,900)	757(41.3)	1,965(1,914 to 2,015)

In Changshu and Yongchuan, 274 (83.3%) doctors and 381 (66.8%) doctors, respectively, had pensions. In contrast, only 8 (2.3%) village doctors in Mianzhu, 9 (3.2%) village doctors in Jingning and 85 (28.1%) village doctors in Liyang had pensions. As a follow-up question, we asked: ‘What is your expected pension per month after you retire?’. The self-reported pensions are shown in Table 5. Large gaps were observed between doctors from different counties. The expected pension was 2,051 RMB per month in Changshu and Liyang, 1,591 RMB per month in Mianzhu, 2,420 RMB per month in Yongchuan, and 1,177 RMB per month in Jingning.

## Discussion

Due to the continuing expansion of the medical education in China [39], the aging of the healthcare workforce should not be a serious problem. In 2011, 68.3% of the licensed (assistant) doctors in hospitals and 73.0% of the licensed (assistant) doctors in the THCs were under 45 years of age; only 4.8% of the licensed (assistant) doctors in the hospitals and 3.2% of the licensed (assistant) doctors in the THCs were over 60 years of age according to the survey by the MOH [24]. However, among the village doctors in our sample, 41.3% were younger than 45 years old, and 30.0% were more than 60 years old, which was consistent with other studies [40,41]. The aging of the village doctors was partly due to the historical causes, as 45.3% of the village doctors were the barefoot doctors who started to work as a doctor before 1985, when the MOH began to use ‘Village Doctor’ to replace ‘Barefoot Doctor’ [16]. As shown above, there was no national pension for the village doctors, so they had to practice at the village clinics until reaching 60 years old or even after 60 years old to earn a living. Even worse, few young medical graduates supplemented the rural health workforce due to low salary and fewer opportunities *etcetera*. As a consequence, the percentage of aging village doctors has increased.

Furthermore, counties with greater economic development had a higher possibility of having older village doctors. As shown in Table 2, the village doctors in Changshu and Liyang were much older than those in Jingning. A potential explanation was that the young in Changshu and Liyang had better chances for making money, whereas practicing medicine in Jingning was not a bad option, which attracted more young people to join the healthcare workforce in the underdeveloped areas.

Unlike the aging problem that existed in all counties, the gender imbalance might only be a problem in poor counties. In Changshu, Liyang, Mianzhu, and Yongchuan, the gender imbalance was not a real problem because female village doctors composed one-third of all village doctors, while in Jingning, only 6.3% of the doctors were female (male/female = 15.0). That was troublesome because most of the public healthcare services (including maternal and gynecological care) were delivered by the village doctors; unfortunately, customs often prevented women from seeking maternal and gynecological care from male village doctors [42].

In general, the western medicine, TCM and mixed methods were applied in all five counties, and mixed methods were the most frequently reported (58.5%), followed by the western medicine (38.5%) and TCM (3.0%). However, the constitutions of the practice methods in the sampled counties differed from each other. The village doctors in Changshu and Liyang were more likely to practice western medicine (44.6%), while the village doctors in Jingning preferred mixed methods (77.5%). This might be due to the different purchasing power of the rural residents in different counties; the GDP per capita in Changshu can be as high as 96,518 RMB, which is 26-fold greater than the 3,711 RMB GDP per capita in Jingning in 2010 [34,37]. The village doctors in Changshu could provide more expensive and profitable western medical services to the consumers, while the village doctors in Jingning preferred mixed methods, which included less expensive TCM.

Similar to the results of the national statistics of village doctors, only 5.3% of the village doctors obtained a degree from junior college or higher, and 75.6% had only completed a secondary school education [24]. The proportion of village doctors with a junior college degree or higher was much lower than that for licensed (assistant) doctors in hospitals (8.8% versus 79.4%) and the licensed (assistant) doctors in THCs (8.8% versus 51.5%) [24]. It might be difficult to meet the goals set by the central government, which requires that all village doctors to have at least a secondary school degree by the end of the year 2015. Only 68.5% of village doctors had met that requirement in the year 2011.

We also found that the practice method was not correlated with the education level (*P* = 0.43), but closely related to the way of obtaining their highest education degree (*P* <0.01). Most of the curricula in China’s medical school were set for western medicine. Therefore, the village doctors who obtained their highest degree from medical school before beginning to work were more likely to practice western medicine, while the village doctors obtaining their highest degree through on-the-job training were more likely to practice mixed methods, as the mixed methods have been used to train the village doctors since the 1970s.

Good remuneration was regarded as a critical incentive for the recruitment and retention of healthcare workers in rural and remote areas [5,7,42-44], and it was also the major dynamic of the push-pull process of the domestic and international healthcare workforce migration [45,46]. The average annual income of the physicians in our study was 21,804 RMB in 2011, which was almost equal to the disposable income of the urban residents (21,810 RMB) and three-fold greater than the net per capita income of the rural residents (6,977 RMB) in 2011 [47]. If one takes the public health service bonus and other incomes from agriculture and other activities (for example, some village doctors have part-time jobs as couriers or drivers) into consideration, the total income for village doctors should be much higher than the income for common rural residents.

However, there are extremely large income gaps among village doctors in different districts. The highest income in our study was 40,000 RMB per month, while 74 village doctors earned less than 200 RMB per month. Actually, village doctors in rural China acted as private practitioners [14] and competed with other village doctors, private clinics and THCs after the collective economy collapsed, and their income was decided by the technique, relative advantages and local economic development. So it should not be surprising that huge income gaps exist among village doctors.

Being private practitioners rather than employees of the government, the village doctors could not enjoy the social protection provided by the government. In essence, the village doctors were treated almost the same as the farmers, although village doctors earned their living by providing medical services to rural residents. Rural residents in China lacked Social Pension Insurance before the State Council launched the New Rural Social Pension Insurance System (NRSPIS) in rural areas in 2009 [48]. Therefore, the village doctors, like the rural residents, could receive 55 RMB per month, but they could not enjoy the additional pension provided by the government for their medical professional service at a national level. In this study, additional pensions were provided to the village doctors only in Changshu and Yongchuan, an effort intended to address the retirement of aging village doctors. Obviously, counties without additional pensions for older doctors might face these same challenges in the future, regardless of the local economic status.

This study, as a longitudinal study, might provide some fundamental knowledge on the current rural health workforce of China. However, a number of limitations of this study should be acknowledged. First, we did not test the validity or reliability of the questionnaires; however, the questionnaires were modified from the well-tested official ones, revised on the basis of focus group input, and their validity was improved with in-depth interviews and pilot surveys. Additionally, not all village doctors participated in this survey, and missing values existed in the data collection. However, 88% was a relatively high response rate, and missing values were very small and randomized. Last, but not least, the generalization of the results should be conservative because it was not a random representative sample of the population.

In the next phase of this study, Hailun County from Heilongjiang Province (northeast China) will be added into this project and more detailed study about the aging, training, and public health services of village doctors will be conducted. Furthermore, physical and mental health conditions of village doctors will also be investigated by survey.

## Conclusions

Gender imbalance of the village doctors, especially in the developed counties, was a serious problem that will be detrimental for the development of rural health. Therefore specific policy to recruit and retain female village doctors will be needed in the future. Low education levels of the village doctors, as a barrier to achieve the educational goals for the village doctors set by the government, should also be tackled through further on-the-job training or by recruiting more college medical graduates into the village clinics. The aging of the village doctors, together with the lack of pension for their professional service, has become an imperative issue, which is critical for the entrance and exit of the rural health workforce. This longitudinal study will track the inner structure of and dynamic evolution of the health workforce, which is meaningful for the development of the rural health workforce. Because health reform in China is still underway, both the positive and negative factors affecting health professionals are decisive to the outcome of the health reform. This longitudinal study will track the health workers’ reactions to health reform, especially in regard to their desire that decision makers make a better and more suitable health reform scheme. In other words, this study will probably show the interplay between the health workforce and the ongoing health reform.

## Abbreviations

GDP: Gross domestic product; MOH: Ministry of Health; TCM: Traditional Chinese Medicine; THCs: Township health centers

## Competing interests

The authors declare that they have no competing interests.

## Authors’ contributions

DT, XZ, ZQ, XW, ZS, HX and WZ participated in the research design and project implementation. HX and WZ, YL, SZ, XQ, participated in the data collection and data analysis. HX and WZ wrote the original text. All of the authors read and approved the final manuscript.
